# Distribution of under-5 deaths in the neonatal, postneonatal, and childhood periods: a multicountry analysis in 64 low- and middle-income countries

**DOI:** 10.1186/s12939-021-01449-8

**Published:** 2021-04-26

**Authors:** Zhihui Li, Omar Karlsson, Rockli Kim, S. V. Subramanian

**Affiliations:** 1 Vanke School of Public Health, Tsinghua University, Beijing, 100084 China; 2 Department of Social and Behavioral Sciences, Harvard T.H. Chan School of Public Health, 677 Huntington Avenue, Boston, MA 02115 USA; 3 Takemi Program in International Health, Harvard T.H. Chan School of Public Health, 677 Huntington Avenue, Boston, MA 02115 USA; 4 Department of Economic History, School of Economics and Management, Lund University, P.O. Box 7083, 220 07 Lund, Sweden; 5 Division of Health Policy & Management, College of Health Science, Korea University, 145 Anam-ro, Seongbuk-gu, Seoul, 02841 South Korea; 6 Interdisciplinary Program in Precision Public Health, Department of Public Health Sciences, Graduate School of Korea University, Seoul, 02841 South Korea; 7 Harvard Center for Population and Development Studies, 9 Bow Street, Cambridge, MA 02138 USA

**Keywords:** Low- and middle-income countries, Distribution of under-5 deaths, Heterogeneity across countries

## Abstract

**Background:**

As under-5 mortality rates declined all over the world, the relative distribution of under-5 deaths during different periods of life changed. To provide information for policymakers to plan for multi-layer health strategies targeting child health, it is essential to quantify the distribution of under-5 deaths by age groups.

**Methods:**

Using 245 Demographic and Health Surveys from 64 low- and middle-income countries conducted between 1986 and 2018, we compiled a database of 2,437,718 children under-5 years old with 173,493 deaths. We examined the share of deaths that occurred in the neonatal (< 1 month), postneonatal (1 month to 1 year old), and childhood (1 to 5 years old) periods to the total number of under-5 deaths at both aggregate- and country-level. We estimated the annual change in share of deaths to track the changes over time. We also assessed the association between share of deaths and Gross Domestic Product (GDP) per capita.

**Results:**

Neonatal deaths accounted for 53.1% (95% confidence interval [CI]: 52.7, 53.4) of the total under-5 deaths. The neonatal share of deaths was lower in low-income countries at 44.0% (43.5, 44.5), and higher in lower-middle-income and upper-middle income countries at 57.2% (56.8, 57.6) and 54.7% (53.8, 55.5) respectively. There was substantial heterogeneity in share of deaths across countries; for example, the share of neonatal to total under-5 deaths ranged from 20.9% (14.1, 27.6) in Eswatini to 82.8% (73.0, 92.6) in Dominican Republic. The shares of deaths in all three periods were significantly associated with GDP per capita, but in different directions—as GDP per capita increased by 10%, the neonatal share of deaths would significantly increase by 0.78 percentage points [PPs] (0.43, 1.13), and the postneonatal and childhood shares of deaths would significantly decrease by 0.29 PPs (0.04, 0.54) and 0.49 PPs (0.24, 0.74) respectively.

**Conclusions:**

Along with the countries’ economic development, an increasing proportion of under-5 deaths occurs in the neonatal period, suggesting a need for multi-layer health strategies with potentially heavier investment in newborn health.

**Supplementary Information:**

The online version contains supplementary material available at 10.1186/s12939-021-01449-8.

## Background

The Millennium Development Goal (MDG) era witnessed an impressive decline in the number of global under-5 child deaths, which were reduced by 53% from 12.7 million to 5.9 million deaths between 1990 and 2015 [1]. However, many countries still fell short of the MDG 4 which targeted a two-thirds reduction in under-5 mortality during this period [2]. The progress was also uneven across age groups as the reduction in neonatal (in the first month of life) deaths declined slower than deaths of older children [3]. Between 1990 and 2015, neonatal deaths reduced by 39% from 4.4 million to 2.7 million, compared to a reduction of more than 60% in postneonatal (between 1 month and 1 year old) and childhood (between 1 and 5 years old) deaths [4, 5].

Under-5 deaths occurring at different ages have different causes that, correspondingly, require different interventions [6–8]. Several multicountry studies have disaggregated deaths of children under-5 years old by age group, examining the mortality rates in neonatal, postneonatal, and childhood periods, separately [9–12]. The importance of disaggregating deaths by age group was also reflected in the Sustainable Development Goals (SDGs) where reducing neonatal mortality (SDG 3.2.2) to 12 deaths per 1000 livebirths, was explicitly listed as one of the goals, separately from reducing under-5 mortality (SDG 3.2.1) to 25 deaths per 1000 livebirths [13].

However, existing studies and major health agendas have almost exclusively focused on the mortality rate indicators [9–12]. Mortality rate, although important in presenting the absolute likelihood of dying at each age, does not provide a clear insight into how the burden of under-5 deaths is distributed by age, and how that distribution changes over time. Exceptions are UNICEF reports that found that under-5 deaths were increasingly concentrated in the neonatal period [14–16]. Yet, these reports were limited to regional-level analyses, which are insufficient to reflect important heterogeneity across countries.

For governments to establish multi-layered health strategies with proper sets of interventions to reduce under-5 deaths, it is essential to examine which age groups have the highest burden of deaths, how the relative burden has changed over time, and how it varies by level of national income. This paper seeks to estimate the distribution of the burden of deaths using the “share” indicators; specifically, we will estimate the share of under-5 deaths occurring in the neonatal, postneonatal, and childhood periods. To our knowledge, the “share” indicator was first used in the field of health two decades ago, but has been applied only in a couple of studies which identified the burden of deaths attributable to non-communicable diseases, compared to communicable diseases, among various population groups [17–19]. Globally, the share of deaths from non-communicable diseases has been rising, particularly in rich countries, with a corresponding shift in intervention emphasis. Similarly, the increased concentration of under-5 deaths in the neonatal period might also call for increased attention to mitigate the causes of neonatal deaths. While the importance of this shift in emphasis is not disputed, an understanding of the variation in the share of under-5 deaths occurring at different ages between countries and over time needs to be established first, in order to tailor policy response and resources appropriately, since neonatal, postneonatal, and childhood deaths may have somewhat different underlying causes.

In this study, besides estimating the share of under-5 deaths occurring in the neonatal, postneonatal, and childhood periods respectively, we analyze how the shares of under-5 deaths in the respective ages varied according to the level of economic development, and how it has shifted over time. The current paper provides by far the most comprehensive picture of the levels and trends in the distribution of under-5 deaths.

## Methods

### Data sources

This study used data from the nationally representative Demographic and Health Survey (DHS) conducted between 1986 and 2018 in 64 low- and middle-income countries (Additional file 1: Table 1)**.** The DHS has been widely adopted as a reliable data source for measuring mortality among children under-5 years old across developing countries [20–22]. The DHS uses extensive interviewer training, standardized measurement tools and techniques, an identical core questionnaire, and instrument pretesting to ensure standardization and comparability across diverse sites and time [20, 23]. The DHS adopts a multistage stratified sampling design, with the first stage generally involving choosing geographically-defined units such as villages or neighborhoods, and the second stage involving selecting the specific households or persons to be interviewed [23, 24]. Details on survey sampling, data collection, and data processing can be found in the countries’ final reports, available from the Measure DHS website [23].

**Table 1:** Share of neonatal, postneonatal, and childhood to total under-5 deaths at aggregate-level, latest survey rounds

	Neonatal	Postneonatal	Childhood
**All available countries**	53.1 (52.7, 53.4)	28.4 (28.1, 28.8)	18.5 (18.2, 18.9)
***By income class***			
Low-income countries	44.0 (43.5, 44.5)	32.8 (32.3, 33.3)	23.2 (22.7, 23.7)
Lower-middle-income countries	57.2 (56.8, 57.6)	26.1 (25.7, 26.5)	16.7 (16.3, 17.1)
Upper-middle-income countries	54.7 (53.8, 55.5)	33.0 (32.2, 33.9)	12.3 (11.6, 13.0)

The GDP per capita used in the analysis was corrected for purchasing power parity and was in constant 2011 US$ and were obtained from the World Bank [25, 26]. For each country, we adopted the GDP per capita of their survey year. We identified whether the country was a low-income country (LIC), a lower-middle-income country (LMIC), or a upper-middle income country (UMIC) in the survey year according to the World Bank classification [27].

This project used publicly-accessible secondary data obtained from the DHS website (https://dhsprogram.conm/data/available-datasets.cfm). The DHS data are not collected specifically for this study and no one on the study team has access to identifiers linked to the data. These activities do not meet the regulatory definition of human subjects research. As such, IRB review is not required. The Harvard Longwood Campus IRB allows researchers to self-determine when their research does not meet the requirements for IRB oversight via the online IRB Decision Tool.

### Study population and sample size

This study restricted the analysis to children born alive within 5 years before the interview. We excluded all surveys with 0 recorded deaths in any period of life (i.e. Armenia 2010, Armenia 2015, Colombia 1986, Moldova 2005, and Vietnam 2002). We also excluded countries with the latest surveys conducted before 2000 to ensure the information were up to date. In total, we included 245 DHS surveys for 64 countries between 1986 and 2018. Our analysis involved a total of 2,437,718 children under-5 years old. We identified a total of 173,493 deaths, with 73,459 of them occurring in the neonatal period, 58,219 occurring in the postneonatal period, and 41,815 occurring in the childhood period.

### Measures

Estimating the share of deaths in each age group allowed us to investigate the distribution of under-5 deaths across ages. In this study, we involved three indicators to measure share of deaths in each period, including share of neonatal to total under-5 deaths (or “neonatal share of deaths”), share of postneonatal to total under-5 deaths (or “postneonatal share of deaths”), and share of childhood to total under-5 deaths (or “childhood share of deaths”), defined as the percentage of under-5 deaths occurring in the neonatal, postneonatal, or childhood period for a certain cohort.

### Statistical analysis

We examined the share of deaths at both aggregate- and country-levels. For analysis at country-level, we examined the latest shares of deaths in 64 countries using the most recent DHS data since 2000. The mean of the latest years was 2013. The median was 2014, with interquartile ranges between 2012 and 2016. We used “stset” command in Stata version 14.2 to trace the number of deaths during the neonatal, postneonatal, and childhood periods for a synthetic cohort based on a reference period of 5 years preceding the survey. We applied DHS sampling weights in the analysis. Following previous practice, we used the bootstrap method by drawing 1000 samples to produce the standard errors and 95% confidence intervals (CIs) for the point estimates [28, 29].

We also tracked the changes in share of deaths over time for each country with available data in multiple survey years. Among the 64 countries involved in the study, we identified 50 countries with multiple surveys conducted between 1986 and 2018. The mean of the gaps between the latest and the earliest surveys was 20 years. The median was 21 years and an interquartile range of 16 and 26 years. We generated annual change in neonatal, postneonatal, and childhood share of deaths, as well as the 95% CIs using the lincom post-estimation commands in Stata.

For analyses at aggregate-level, we pooled the observations from the latest surveys for the 64 countries. We followed previous practice [30] and reweighted the observations in each survey in proportion to the country’s population size at the time of survey. The population size was obtained from the World Bank data [31]. The rest of the procedure to generate the point estimates and 95% CIs of the share of deaths was the same as at country-level.

## Results

### Aggregate-level share of deaths

Using pooled data from 64 countries, we show the share of deaths in each period in Table 1. We found that among all under-5 deaths, 53.1% (95% CI: 52.7, 53.4) occurred in the neonatal period, 28.4% (95% CI: 28.1, 28.8) were in the postneonatal period, and 18.5% (95% CI: 18.2, 18.9) were in the childhood period. Countries with higher income appeared to have significantly larger neonatal share of deaths – in LICs, 44.0% (95% CI: 43.5, 44.5) of the under-5 deaths happened in the neonatal period, compared to 57.2% (95% CI: 56.8, 57.6) in LMICs and 54.7% (95% CI: 53.8, 55.5) in UMICs. On the other hand, childhood share of deaths decreased in countries with higher income level – in LICs, 23.2% (95% CI: 22.7, 23.7) of the under-5 deaths occurred in the childhood period, which reduced to 16.7% (95% CI: 16.3, 17.1) in LMICs, and further reduced to 12.3% (95% CI: 11.6, 13.0) in UMICs (Table 1). When we narrowed down the countries with the latest surveys conducted between 2008 and 2018 (Additional file 1: Table 2) or included the countries with 0 recorded deaths in any period of life (Additional file 1: Table 3), the results remained similar.

### Country-level share of deaths, latest years

We presented the latest share of deaths in each country in Table 2. The neonatal share of deaths ranged widely from as low as 20.9% (95% CI: 14.1, 27.6) in Eswatini to as high as 82.8% (95% CI: 73.0, 92.6) in Dominican Republic; similarly, the postneonatal share of deaths ranged from 5.2% (95% CI: − 0.6, 10.9) in Dominican Republic to 59.0% (95% CI: 50.8, 67.2) in Eswatini; and the childhood share of deaths ranged from 2.8% (95% CI: − 2.7, 8.2) in Guyana to 43.4% (39.3, 47.4) in Niger.

**Table 2:** Share of neonatal, postneonatal, and childhood to total under-5 deaths, and their ranks among 64 countries, latest survey rounds

Country	Year	Income	Neonatal to total under-5 deaths		Postneonatal to total under-5 deaths		Childhood to total under-5 deaths	
			Share (%)	Rank	Share (%)	Rank	Share (%)	Rank
Dominican Republic	2013	UM	82.8 (73.0, 92.6)	1	5.2(−0.6, 10.9)	64	12.1 (3.6, 20.5)	41
Kyrgyzstan	2012	L	77.6 (65.7, 89.4)	2	14.3 (4.4, 24.2)	63	8.2 (0.4, 15.9)	57
Morocco	2003	LM	72.4 (63.6, 81.3)	3	21.4 (13.3, 29.6)	60	6.1 (1.4, 10.9)	63
Honduras	2011	LM	69.9 (61.0, 78.8)	4	19.4 (11.7, 27.1)	61	10.7 (4.7, 16.7)	47
Turkey	2008	UM	68.0 (49.3, 86.7)	5	16.0 (1.3, 30.7)	62	16.0 (1.3, 30.7)	38
Bangladesh	2014	LM	67.5 (60.1, 75.0)	6	23.4 (16.7, 30.1)	55	9.1 (4.5, 13.6)	53
India	2015	LM	66.4 (65.1, 67.7)	7	23.3 (22.1, 24.4)	56	10.3 (9.5, 11.1)	50
Azerbaijan	2006	LM	65.5 (53.2, 77.9)	8	25.9 (14.5, 37.2)	50	8.6 (1.3, 15.9)	55
Armenia	2005	LM	65.5 (47.9, 83.1)	9	27.6 (11.0, 44.1)	45	6.9(−2.5, 16.3)	60
Jordan	2017	UM	64.2 (54.5, 73.9)	10	29.5 (20.3, 38.7)	38	6.3 (1.4, 11.2)	62
Cambodia	2014	L	64.0 (55.2, 72.9)	11	25.4 (17.4, 33.5)	52	10.5 (4.9, 16.2)	49
Pakistan	2017	LM	63.7 (59.1, 68.3)	12	28.0 (23.7, 32.3)	43	8.3 (5.7, 11.0)	56
Senegal	2017	L	62.9 (56.3, 69.6)	13	25.9 (19.8, 31.9)	51	11.2 (6.9, 15.6)	46
Ghana	2014	LM	61.9 (52.6, 71.2)	14	21.9 (14.0, 29.9)	58	16.2 (9.1, 23.3)	37
Guyana	2009	LM	61.1 (45.0, 77.3)	15	36.1 (20.2, 52.0)	15	2.8(−2.7, 8.2)	64
Philippines	2017	LM	61.0 (52.2, 69.9)	16	27.1 (19.1, 35.2)	47	11.9 (6.0, 17.7)	42
Peru	2012	UM	59.4 (47.7, 71.1)	17	30.4 (19.5, 41.4)	32	10.1 (3.0, 17.3)	51
Yemen	2013	LM	59.3 (54.2, 64.3)	18	29.2 (24.6, 33.9)	39	11.5 (8.2, 14.7)	44
Colombia	2015	UM	59.0 (48.0, 70.0)	19	32.1 (21.6, 42.5)	27	9.0 (2.6, 15.4)	54
Nepal	2016	L	56.5 (46.3, 66.7)	20	37.0 (27.0, 46.9)	9	6.5 (1.4, 11.6)	61
Guatemala	2014	LM	56.2 (49.2, 63.3)	21	32.3 (25.7, 38.9)	26	11.5 (6.9, 16.0)	45
Indonesia	2017	LM	56.2 (49.8, 62.6)	22	27.5 (21.7, 33.2)	46	16.3 (11.6, 21.1)	36
Bolivia	2008	LM	55.4 (49.2, 61.5)	23	36.7 (30.7, 42.6)	11	8.0 (4.6, 11.3)	58
Egypt	2014	LM	55.2 (48.6, 61.7)	24	35.0 (28.7, 41.3)	17	9.9 (5.9, 13.8)	52
Malawi	2015	L	54.6 (50.0, 59.2)	25	26.5 (22.4, 30.6)	49	18.9 (15.2, 22.5)	30
Comoros	2012	L	54.5 (43.4, 65.7)	26	28.6 (18.4, 38.7)	41	16.9 (8.5, 25.3)	35
Myanmar	2015	LM	53.2 (43.1, 63.3)	27	36.2 (26.4, 45.9)	13	10.6 (4.4, 16.9)	48
Uganda	2016	L	52.7 (47.3, 58.2)	28	28.7 (23.8, 33.6)	40	18.6 (14.4, 22.8)	31
Timor-Leste	2016	LM	52.7 (44.1, 61.4)	29	23.3 (15.9, 30.6)	57	24.0 (16.6, 31.4)	15
Gambia	2013	L	51.6 (44.4, 58.9)	30	33.0 (26.1, 39.8)	23	15.4 (10.1, 20.6)	40
Gabon	2012	UM	51.3 (43.3, 59.3)	31	21.7 (15.1, 28.3)	59	27.0 (19.9, 34.1)	10
Ethiopia	2016	L	51.1 (46.3, 55.8)	32	30.4 (26.0, 34.8)	33	18.5 (14.8, 22.2)	32
Angola	2015	UM	50.6 (45.3, 55.9)	33	29.7 (24.8, 34.6)	37	19.7 (15.5, 23.9)	27
Zimbabwe	2015	L	50.5 (43.8, 57.1)	34	30.1 (24.0, 36.2)	35	19.4 (14.2, 24.7)	28
Papua New Guinea	2017	LM	49.1 (42.5, 55.7)	35	32.9 (26.7, 39.1)	24	18.0 (13.0, 23.1)	34
Lesotho	2014	LM	49.0 (39.4, 58.7)	36	31.7 (22.7, 40.7)	28	19.2 (11.6, 26.8)	29
Kenya	2014	LM	48.7 (44.2, 53.2)	37	35.5 (31.2, 39.9)	16	15.7 (12.4, 19.0)	39
Togo	2013	L	47.7 (40.3, 55.1)	38	27.6 (20.9, 34.2)	44	24.7 (18.3, 31.1)	14
Rwanda	2014	L	47.5 (40.1, 54.8)	39	31.3 (24.5, 38.1)	29	21.2 (15.2, 27.2)	24
Haiti	2016	L	47.1 (40.5, 53.7)	40	34.5 (28.3, 40.8)	18	18.4 (13.3, 23.5)	33
Afghanistan	2015	L	46.9 (43.5, 50.2)	41	45.4 (42.1, 48.8)	2	7.7 (5.9, 9.5)	59
Tanzania	2015	L	46.6 (40.6, 52.6)	42	31.2 (25.6, 36.8)	30	22.2 (17.2, 27.2)	20
Sao Tome and Principe	2008	LM	46.5 (31.4, 61.6)	43	30.2 (16.3, 44.1)	34	23.3 (10.5, 36.0)	18
Mali	2018	L	44.8 (40.1, 49.6)	44	24.4 (20.3, 28.5)	53	30.8 (26.4, 35.1)	5
Nicaragua	2001	L	44.8 (35.2, 54.3)	45	33.3 (24.3, 42.4)	20	21.9 (14.0, 29.9)	21
Côte d’Ivoire	2011	LM	44.5 (38.8, 50.2)	46	33.2 (27.8, 38.6)	21	22.3 (17.5, 27.0)	19
Tajikistan	2017	L	44.1 (34.9, 53.4)	47	44.1 (34.9, 53.4)	3	11.7 (5.7, 17.7)	43
Congo	2011	LM	44.1 (37.5, 50.7)	48	26.8 (21.0, 32.7)	48	29.1 (23.1, 35.1)	8
Zambia	2013	LM	43.4 (38.5, 48.2)	49	33.1 (28.5, 37.7)	22	23.6 (19.4, 27.7)	16
Benin	2017	L	43.0 (38.5, 47.5)	50	29.9 (25.7, 34.0)	36	27.1 (23.1, 31.1)	9
Madagascar	2008	L	42.6 (37.4, 47.8)	51	36.9 (31.9, 42.0)	10	20.5 (16.2, 24.7)	25
Nigeria	2018	LM	42.0 (39.6, 44.4)	52	24.0 (21.9, 26.1)	54	34.0 (31.7, 36.4)	2
Liberia	2013	L	38.3 (31.7, 44.9)	53	32.5 (26.2, 38.9)	25	29.2 (23.0, 35.4)	7
Burundi	2016	L	38.1 (33.7, 42.6)	54	37.0 (32.6, 41.5)	8	24.8 (20.9, 28.8)	13
Mozambique	2011	L	37.6 (33.0, 42.3)	55	41.0 (36.3, 45.7)	6	21.3 (17.4, 25.3)	23
Democratic Republic of the Congo	2013	L	37.6 (34.0, 41.2)	56	36.3 (32.7, 39.9)	12	26.1 (22.8, 29.4)	11
Namibia	2013	UM	35.9 (26.6, 45.2)	57	42.7 (33.1, 52.3)	4	21.4 (13.4, 29.3)	22
Cameroon	2011	LM	35.8 (31.6, 40.0)	58	31.0 (27.0, 35.1)	31	33.2 (29.1, 37.3)	3
Chad	2014	L	35.6 (32.6, 38.6)	59	33.7 (30.7, 36.7)	19	30.7 (27.8, 33.6)	6
Guinea	2018	L	35.3 (29.8, 40.8)	60	38.8 (33.1, 44.4)	7	26.0 (20.9, 31.0)	12
Sierra Leone	2013	L	35.2 (31.8, 38.5)	61	41.5 (38.0, 45.0)	5	23.3 (20.3, 26.3)	17
Burkina Faso	2010	L	31.0 (27.5, 34.4)	62	36.2 (32.6, 39.7)	14	32.9 (29.4, 36.3)	4
Niger	2012	L	28.6 (24.9, 32.2)	63	28.1 (24.4, 31.7)	42	43.4 (39.3, 47.4)	1
Eswatini	2006	LM	20.9 (14.1, 27.6)	64	59.0 (50.8, 67.2)	1	20.1 (13.5, 26.8)	26

1. Income classification: “L” represents “low-income country”; “LM” represents “lower-middle-income country”; “UM” represents “upper-middle-income country”

Among the top 10 countries with the highest neonatal share of deaths, only one country was LIC, which was Kyrgyzstan; however, seven of the bottom 10 countries with the lowest neonatal share of deaths were LICs, suggesting LICs were more likely to have a smaller share of neonatal deaths. On the contrary, eight of the top 10 countries with the highest postneonatal share of deaths and six of the top 10 countries with the highest childhood share of deaths were LICs, while few countries with the lowest postneonatal or childhood share of deaths were LICs, indicating LICs were more likely to have a larger proportion of child deaths concentrated in the postneonatal and childhood periods (Table 2).

**Fig. 1:**
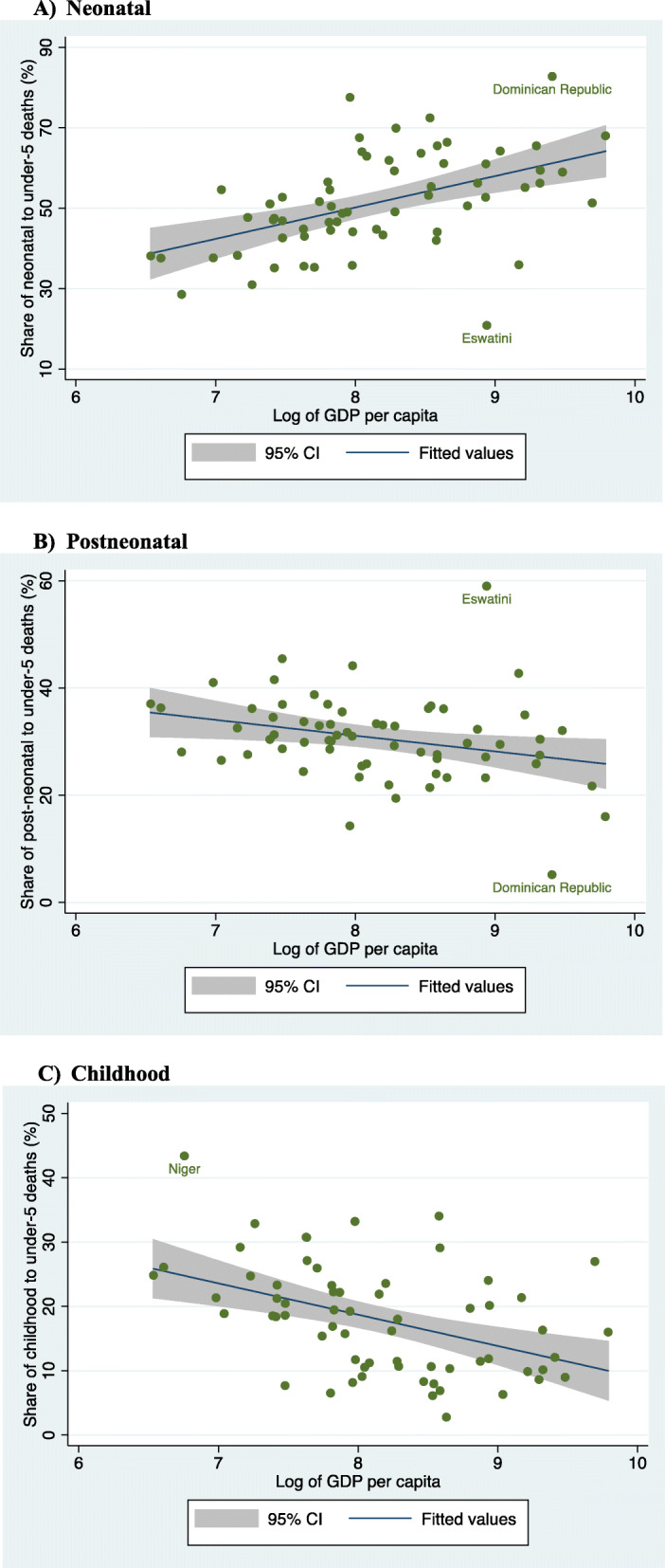
Correlation between GDP per capita (PPP, constant 2011 international $) and share of neonatal, postneonatal, childhood to total under-5 deaths (%), latest survey rounds. **a** Neonatal. **b** Postneonatal. **c** Childhood

In Fig. 1, we further examined the association between countries’ GDP per capita and share of deaths. We found neonatal share of deaths to be significantly and positively associated with GDP per capita – a 10% greater GDP per capita was associated with a 0.78 percentage points [PPs] (95% CI: 0.43, 1.13) increase in neonatal share of deaths (*P* < 0.001). On the other hand, GDP per capita was negatively associated with postneonatal and childhood shares of deaths: a 10% greater GDP per capita was associated with a 0.29 PPs (95% CI: 0.04, 0.54) lower postneonatal share of deaths (*P* < 0.001) and 0.49 PPs (95% CI: 0.24, 0.74) lower childhood share of deaths (*P* < 0.001) .

### Country-level share of deaths over time

**Fig. 2:**
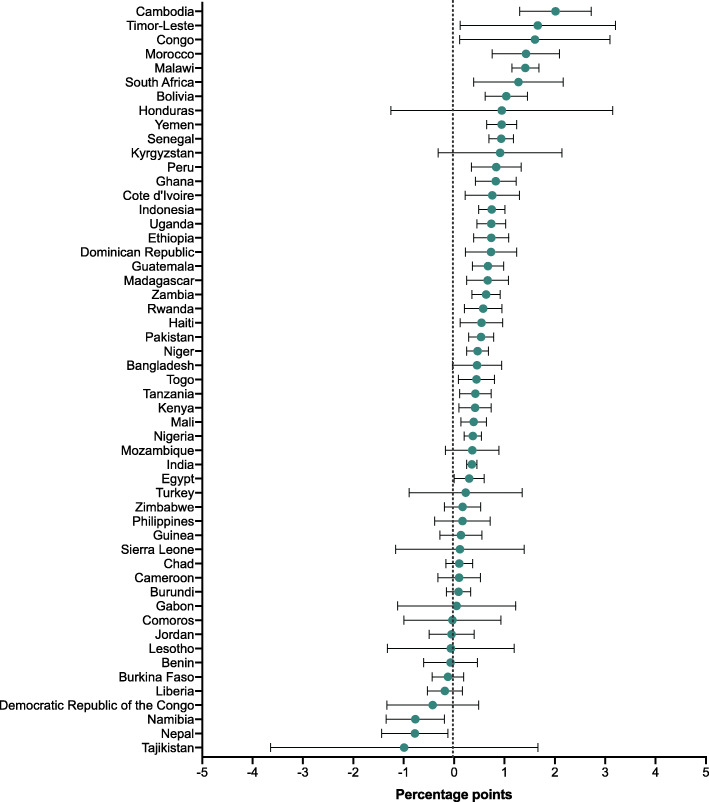
Annual change in share of neonatal to total under-5 deaths (percentage points)

**Fig. 3:**
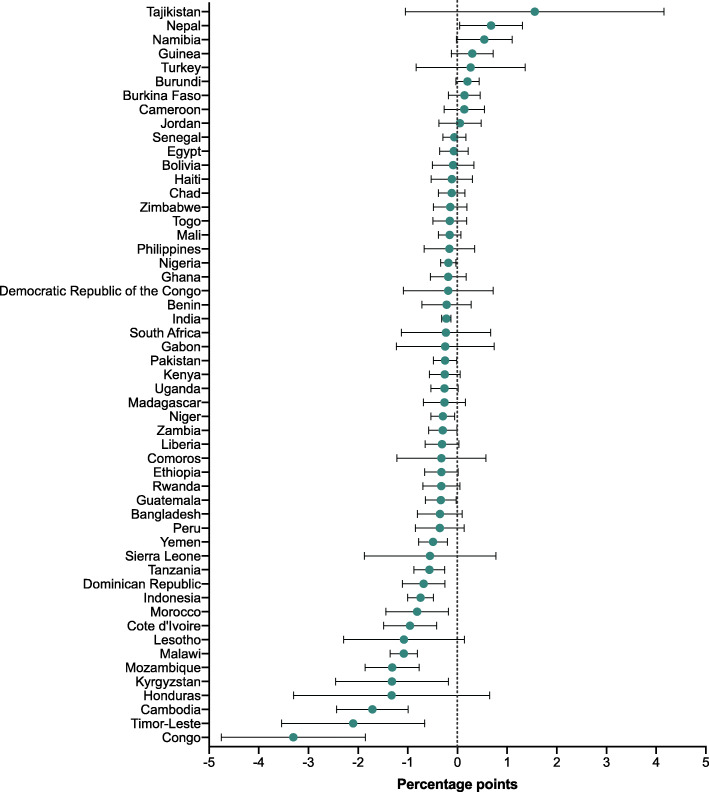
Annual change in share of postneonatal to total under-5 deaths (percentage points)

**Fig. 4:**
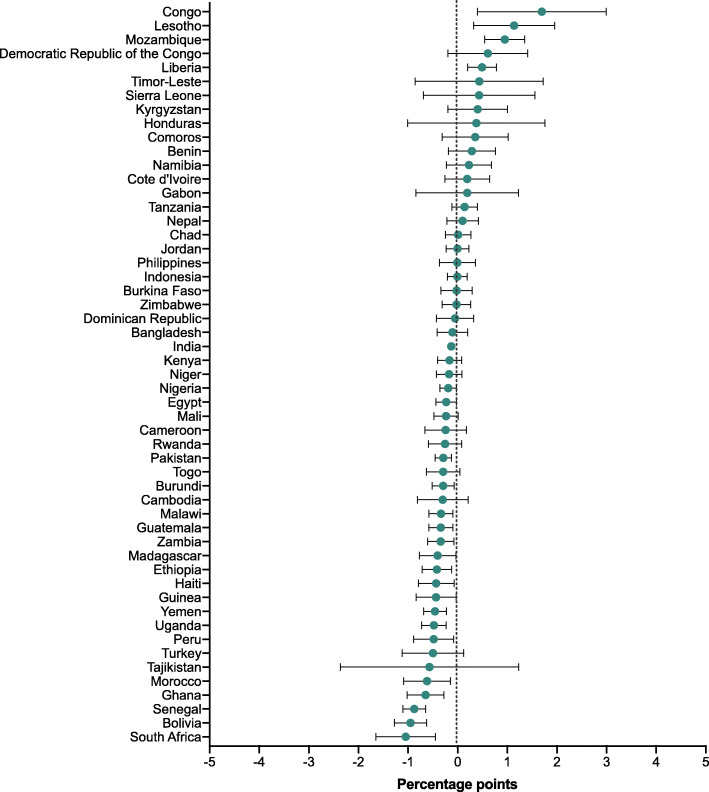
Annual change in share of childhood to total under-5 deaths (percentage points)

For the 50 countries with multiple survey rounds, we examined the change in share of deaths over time in Additional file 1: Tables 4 to 6 and showed the annualized change in Figs. 2, 3 and 4. Among the 50 countries, neonatal share of deaths significantly increased in 28 countries which belonged to various income groups (low-, lower-middle-, or upper-middle- income groups). The annual change increased fastest in Cambodia (2.0 PPs [95% CI: 1.3, 2.7]), followed by Timor-Leste (1.7 [95% CI: 0.1, 3.2]) and Congo (1.6 [95% CI: 0.1, 3.1]. Meanwhile, the neonatal share of deaths only significantly decreased in two countries, which were Namibia, and Nepal at an annual rate of − 0.8 PPs (95% CI: − 1.4, − 0.2) and − 0.8 PPs (95% CI: − 1.4, − 0.1) respectively. Moreover, we found postneonatal share of deaths significantly increased in only one country (Nepal), but significantly decreased in 17 countries; similarly, childhood share of deaths significantly increased in merely four countries (Congo, Lesotho, Liberia, and Mozambique), but decreased in 19 countries of different income groups.

## Discussion

To our knowledge, this study is the first to systematically examine the distribution of under-5 deaths in different periods of life. There were three salient findings of this study: First, at the aggregate-level, more than half of the under-5 deaths occurred in the neonatal period; the neonatal share of deaths was considerably lower in LICs than in LMICs and UMICs, meanwhile, the childhood share of deaths was higher in LICs than the better-off countries. Second, at the country-level, we found substantial heterogeneity across countries with consistent findings as in the aggregate results. Third, under-5 deaths became increasingly concentrated in the neonatal period over time in around half of the studied countries, which was not limited to countries of any certain economic status.

Although there has been increasing interest in examining children’s survival disparately by age group [9–12], our study was the first to provide an intuitive picture of how under-5 deaths are distributed across various age groups, both at aggregate- and country-levels, and how this distribution varies by level of GDP and over time. Such information is a critical addition to the traditional evidence on mortality rates; for instance, although the evidence on mortality rate showed that Ethiopia reached the MDG 4 target to reduce under-5 mortality rate by two thirds 3 years before the 2015 deadline [12], it overlooked the fact that the share of neonatal to total under-5 deaths increased from 39% in 2000 to 51% in 2016. The increased proportion was not a bad signal for the country, in fact, it was likely to be a result of the country’s remarkable progress in reducing child deaths in the postneonatal and childhood periods. Yet with the increasing share of neonatal deaths, in order to achieve the SDG 3.2.2 of reducing neonatal mortality to 12 deaths per 1000 live births or less by 2030, we call for more attention to address newborn health in the future development of Ethiopia’s healthcare system. Nepal has performed outstandingly in the MDG era by achieving the MDG 4 in 2010 and was among the only two countries with a decreasing neonatal share of deaths [32]. The accomplishment can be largely attributed to Nepal’s multi-layered health strategies, which started from promoting reproductive, maternal and child services in 1980s, and gradually extended its focus to neonatal health in 2000s. Nepal’s health care system heavily relied on the community-based health network composed of community-based health workers (CHWs), female community health volunteers, traditional birth attendants, and the civil society to reach the most disadvantaged population [33, 34]. Based on this well-functioning community-based health system, in 2004, Nepal adopted its National Neonatal Health Strategy, and later in 2007, further adopted the Community-Based Newborn Care Program, which delivered essential perinatal and newborn care through the community health network [34, 35]. Various other interventions to reduce neonatal deaths have also been addressed in previous literatures [32, 36], such as sufficient antenatal care, institutional delivery, delivery with skilled birth attendants, and timely postnatal check for newborns. Recent evidence from low- and middle-income countries in Africa, Asia and Latin America also pointed to the importance of quality of care, adherence to health guidelines, access to adequate inter-facility transport for emergency obstetric care, and availability of neonatal intensive care [32, 37, 38].

Our work by no means discourage investment in postneonatal and childhood health. Actually, we believe that neonatal, postneonatal and childhood health could be promoted in an integral way, despite the recognized differences in various child development periods. For countries with higher burden of deaths concentrated among older children (e.g. *Niger* and Nigeria), interventions such as prevention and treatment of pneumonia and diarrhea, complementary feeding, immunizations, nutritional supplementation, improvement in hygiene, and case management of HIV and malaria showed salient effects on children’s survival [6–8]. Many of these interventions could be carried out by the community health network or via community-based activities [39, 40]. For example, CHWs were found to be effective in different types of prevention interventions, such as malaria prevention, health education, breastfeeding promotion, essential newborn care and psychosocial support, which are cited as some of the most cost-effective ways to save lives [41, 42].

This study has several limitations that are mostly related to the comprehensiveness and validity of the secondary data we adopted. First, we were only able to include 64 low- and middle- income countries with available data. Therefore, the aggregate-level estimates were not representative at either global-level or by income groups. Second, due to the varying availability of data in the DHS by country, we were able to conduct a trend analysis only for a subset of countries, which limited our knowledge on how the share of deaths changed over time in developing countries. With more data available in the future, the aggregate-level analysis could be strengthened. Third, although DHS has been widely adopted as the most reliable source on child mortality [9–11, 22], we recognize the potential data collection problems, including misreporting dates to birth or misreporting age at death [43].

## Conclusions

Despite these limitations, this was the first study providing a comprehensive picture on the distribution of child deaths in each period of child development. Neonatal deaths accounted for around half of all under-5 deaths, and appeared to be more concentrated in more affluent countries. Along with the countries’ economic development, an increasing proportion of under-5 deaths occurs in the neonatal period, suggesting a need for multi-layer health strategies with potentially heavier investment in newborn health, yet country-specific context should be considered in national policy discussions. Future research should explore a detailed assessment of cost-effective interventions to alleviate death burden in each period, and guide the countries to develop multi-layered health strategies that are based on the country’s specific context.

## Supplementary Information

**Additional file 1: Table 1**. Share of neonatal, postneonatal, and childhood to total under-5 deaths. **Table 2.** Share of neonatal, postneonatal, and childhood to total under-5 deaths at aggregate-level, latest survey rounds between 2008 and 2018. **Table 3.** Share of neonatal, postneonatal, and childhood to total under-5 deaths at aggregate-level including the countries with 0 recorded deaths in any period of life, latest survey rounds. **Table 4.** Change in share of neonatal to total under-5 deaths. **Table 5.** Change in share of postneonatal to total under-5 deaths. **Table 6**. Change in share of childhood to total under-5 deaths.

## Data Availability

DHS data are available at https://dhsprogram.com (requiring a simple application).

## Abbreviations

GDP: Gross Domestic Product
PPs: Percentage points
MDG: Millennium Development Goal
SDG: Sustainable Development Goal
DHS: Demographic and Health Survey
LIC: Low-income country
LMIC: Lower-middle-income country
UMIC: Upper-middle income country
