# Supplementary material for: Distribution of under-5 deaths in the neonatal, postneonatal, and childhood periods: a multicountry analysis in 64 low- and middle-income countries
Source: Int J Equity Health. 2021 Apr 26;20:109. doi: 10.1186/s12939-021-01449-8 (PMC8077916; doi:10.1186/s12939-021-01449-8)
Supplement: Supplementary file 1 — Additional file 1: Table 1. Share of neonatal, postneonatal, and childhood to total under-5 deaths. Table 2. Share of neonatal, postneonatal, and childhood to total under-5 deaths at aggregate-level, latest survey rounds between 2008 and 2018. Table 3. Share of neonatal, postneonatal, and childhood to total under-5 deaths at aggregate-level including the countries with 0 recorded deaths in any period of life, latest survey rounds. Table 4. Change in share of neonatal to total under-5 deaths. Table 5. Change in share of postneonatal to total under-5 deaths. Table 6. Change in share of childhood to total under-5 deaths. [file 12939_2021_1449_MOESM1_ESM.docx]

**Additional**

**Additional Table 1. Share of neonatal, postneonatal, and childhood to total under-5 deaths**

| **Country** | **Year** | **Share of deaths (%)** | | |
| --- | --- | --- | --- | --- |
|  |  | **Neonatal** | **Post-neonatal** | **Childhood** |
| Afghanistan | 2015 | 46.9(43.5, 50.2) | 45.4(42.1, 48.8) | 7.7(5.9, 9.5) |
| Angola | 2015 | 50.6(45.3, 55.9) | 29.7(24.8, 34.6) | 19.7(15.5, 23.9) |
| Armenia | 2005 | 65.5(47.9, 83.1) | 27.6(11.0, 44.1) | 6.9(-2.5, 16.3) |
| Azerbaijan | 2006 | 65.5(53.2, 77.9) | 25.9(14.5, 37.2) | 8.6(1.3, 15.9) |
| Bangladesh | 1993 | 57.9(51.0, 64.9) | 30.8(24.3, 37.3) | 11.3(6.8, 15.7) |
| Bangladesh | 1996 | 50.0(45.0, 55.0) | 32.6(27.9, 37.2) | 17.4(13.6, 21.2) |
| Bangladesh | 1999 | 54.9(48.4, 61.4) | 29.5(23.5, 35.4) | 15.6(10.9, 20.4) |
| Bangladesh | 2004 | 56.0(49.8, 62.2) | 29.6(23.9, 35.3) | 14.4(10.0, 18.8) |
| Bangladesh | 2007 | 68.0(60.6, 75.4) | 20.9(14.4, 27.4) | 11.1(6.1, 16.1) |
| Bangladesh | 2011 | 65.2(58.4, 72.1) | 22.5(16.5, 28.5) | 12.3(7.6, 17.0) |
| Bangladesh | 2014 | 67.5(60.1, 75.0) | 23.4(16.7, 30.1) | 9.1(4.5, 13.6) |
| Benin | 1996 | 44.4(34.1, 54.8) | 34.4(24.6, 44.3) | 21.1(12.6, 29.6) |
| Benin | 2001 | 36.4(30.0, 42.7) | 37.3(30.9, 43.7) | 26.4(20.5, 32.2) |
| Benin | 2006 | 37.8(34.0, 41.5) | 35.4(31.8, 39.1) | 26.8(23.4, 30.2) |
| Benin | 2011 | 45.9(39.9, 51.9) | 32.5(26.8, 38.1) | 21.6(16.7, 26.6) |
| Benin | 2017 | 43.0(38.5, 47.5) | 29.9(25.7, 34.0) | 27.1(23.1, 31.1) |
| Bolivia | 1989 | 35.7(30.6, 40.8) | 38.3(33.1, 43.5) | 26.0(21.4, 30.7) |
| Bolivia | 1994 | 43.3(35.7, 50.9) | 40.2(32.7, 47.8) | 16.5(10.8, 22.2) |
| Bolivia | 1998 | 46.4(39.8, 53.0) | 39.2(32.8, 45.6) | 14.4(9.8, 19.0) |
| Bolivia | 2003 | 43.5(37.9, 49.0) | 36.3(30.9, 41.7) | 20.3(15.8, 24.8) |
| Bolivia | 2008 | 55.4(49.2, 61.5) | 36.7(30.7, 42.6) | 8.0(4.6, 11.3) |
| Burkina Faso | 1993 | 33.0(29.0, 37.0) | 33.8(29.7, 37.8) | 33.2(29.2, 37.2) |
| Burkina Faso | 1998 | 26.6(23.3, 30.0) | 38.7(34.9, 42.4) | 34.7(31.1, 38.3) |
| Burkina Faso | 2003 | 24.2(21.1, 27.4) | 33.5(30.0, 37.0) | 42.3(38.6, 45.9) |
| Burkina Faso | 2010 | 31.0(27.5, 34.4) | 36.2(32.6, 39.7) | 32.9(29.4, 36.3) |
| Burundi | 1987 | 35.5(30.3, 40.8) | 31.2(26.1, 36.2) | 33.3(28.2, 38.5) |
| Burundi | 2010 | 41.8(36.2, 47.5) | 36.1(30.6, 41.6) | 22.1(17.4, 26.9) |
| Burundi | 2016 | 38.1(33.7, 42.6) | 37.0(32.6, 41.5) | 24.8(20.9, 28.8) |
| Cambodia | 2000 | 35.8(31.3, 40.4) | 49.4(44.7, 54.2) | 14.8(11.4, 18.1) |
| Cambodia | 2005 | 41.5(35.3, 47.6) | 46.7(40.5, 53.0) | 11.8(7.8, 15.8) |
| Cambodia | 2010 | 61.9(55.0, 68.7) | 28.9(22.5, 35.3) | 9.3(5.2, 13.4) |
| Cambodia | 2014 | 64.0(55.2, 72.9) | 25.4(17.4, 33.5) | 10.5(4.9, 16.2) |
| Cameroon | 1991 | 33.7(26.5, 41.0) | 28.2(21.3, 35.2) | 38.0(30.6, 45.5) |
| Cameroon | 1998 | 35.8(27.3, 44.3) | 37.4(28.8, 46.0) | 26.8(19.0, 34.7) |
| Cameroon | 2004 | 28.8(23.7, 33.8) | 37.6(32.1, 43.0) | 33.7(28.4, 39.0) |
| Cameroon | 2011 | 35.8(31.6, 40.0) | 31.0(27.0, 35.1) | 33.2(29.1, 37.3) |
| Chad | 1996 | 33.7(30.0, 37.4) | 35.8(32.0, 39.5) | 30.5(26.9, 34.1) |
| Chad | 2004 | 27.3(23.4, 31.3) | 40.0(35.7, 44.3) | 32.7(28.5, 36.8) |
| Chad | 2014 | 35.6(32.6, 38.6) | 33.7(30.7, 36.7) | 30.7(27.8, 33.6) |
| Colombia | 1990 | 47.8(27.0, 68.7) | 26.1(7.7, 44.4) | 26.1(7.7, 44.4) |
| Colombia | 1995 | 53.3(40.6, 66.1) | 28.3(16.8, 39.8) | 18.3(8.5, 28.2) |
| Colombia | 2000 | 83.3(69.8, 96.9) | 13.3(1.0, 25.7) | 3.3(-3.2, 9.9) |
| Colombia | 2005 | 65.6(57.5, 73.8) | 26.0(18.4, 33.5) | 8.4(3.6, 13.2) |
| Colombia | 2010 | 71.1(63.4, 78.8) | 18.5(11.9, 25.1) | 10.4(5.2, 15.5) |
| Colombia | 2015 | 59.0(48.0, 70.0) | 32.1(21.6, 42.5) | 9.0(2.6, 15.4) |
| Comoros | 1996 | 55.1(44.7, 65.4) | 33.7(23.8, 43.6) | 11.2(4.6, 17.8) |
| Comoros | 2012 | 54.5(43.4, 65.7) | 28.6(18.4, 38.7) | 16.9(8.5, 25.3) |
| Congo | 2005 | 34.5(28.4, 40.5) | 46.6(40.3, 53.0) | 18.9(13.9, 23.9) |
| Congo | 2011 | 44.1(37.5, 50.7) | 26.8(21.0, 32.7) | 29.1(23.1, 35.1) |
| Côte d'Ivoire | 1994 | 31.6(24.7, 38.5) | 49.4(42.0, 56.9) | 19.0(13.1, 24.8) |
| Côte d'Ivoire | 1998 | 42.7(34.2, 51.3) | 31.3(23.3, 39.3) | 26.0(18.4, 33.5) |
| Côte d'Ivoire | 2011 | 44.5(38.8, 50.2) | 33.2(27.8, 38.6) | 22.3(17.5, 27.0) |
| Democratic Republic of the Congo | 2007 | 40.1(36.0, 44.3) | 37.4(33.4, 41.5) | 22.4(18.9, 25.9) |
| Democratic Republic of the Congo | 2013 | 37.6(34.0, 41.2) | 36.3(32.7, 39.9) | 26.1(22.8, 29.4) |
| Dominican Republic | 1986 | 62.9(55.7, 70.2) | 23.5(17.1, 29.9) | 13.5(8.4, 18.7) |
| Dominican Republic | 1991 | 52.5(42.6, 62.4) | 34.3(24.9, 43.7) | 13.1(6.4, 19.8) |
| Dominican Republic | 1996 | 55.9(46.2, 65.6) | 34.3(25.1, 43.6) | 9.8(4.0, 15.6) |
| Dominican Republic | 2002 | 66.2(59.8, 72.6) | 24.9(19.1, 30.7) | 8.9(5.1, 12.8) |
| Dominican Republic | 2007 | 66.8(60.4, 73.2) | 25.5(19.5, 31.4) | 7.7(4.1, 11.3) |
| Dominican Republic | 2013 | 82.8(73.0, 92.6) | 5.2(-0.6, 10.9) | 12.1(3.6, 20.5) |
| Egypt | 1988 | 47.3(43.2, 51.4) | 36.8(32.9, 40.8) | 15.9(12.9, 18.9) |
| Egypt | 1992 | 49.1(44.0, 54.1) | 35.4(30.5, 40.2) | 15.5(11.9, 19.2) |
| Egypt | 1995 | 48.5(43.3, 53.7) | 41.2(36.1, 46.3) | 10.4(7.2, 13.5) |
| Egypt | 2000 | 52.3(46.7, 57.9) | 37.3(31.9, 42.7) | 10.4(7.0, 13.8) |
| Egypt | 2005 | 59.2(52.5, 65.8) | 31.9(25.6, 38.2) | 8.9(5.1, 12.8) |
| Egypt | 2008 | 66.7(57.5, 75.9) | 26.5(17.9, 35.1) | 6.9(1.9, 11.8) |
| Egypt | 2014 | 55.2(48.6, 61.7) | 35.0(28.7, 41.3) | 9.9(5.9, 13.8) |
| Eswatini | 2006 | 20.9(14.1, 27.6) | 59.0(50.8, 67.2) | 20.1(13.5, 26.8) |
| Ethiopia | 2000 | 39.2(36.3, 42.2) | 35.5(32.7, 38.4) | 25.2(22.6, 27.8) |
| Ethiopia | 2005 | 42.5(38.7, 46.2) | 36.8(33.1, 40.4) | 20.8(17.7, 23.9) |
| Ethiopia | 2011 | 53.1(48.9, 57.3) | 27.9(24.2, 31.7) | 18.9(15.6, 22.2) |
| Ethiopia | 2016 | 51.1(46.3, 55.8) | 30.4(26.0, 34.8) | 18.5(14.8, 22.2) |
| Gabon | 2000 | 50.7(39.1, 62.2) | 24.7(14.7, 34.6) | 24.7(14.7, 34.6) |
| Gabon | 2012 | 51.3(43.3, 59.3) | 21.7(15.1, 28.3) | 27.0(19.9, 34.1) |
| Gambia | 2013 | 51.6(44.4, 58.9) | 33.0(26.1, 39.8) | 15.4(10.1, 20.6) |
| Ghana | 1988 | 40.4(35.8, 44.9) | 26.7(22.6, 30.8) | 33.0(28.6, 37.3) |
| Ghana | 1993 | 60.8(52.9, 68.7) | 27.0(19.8, 34.2) | 12.2(6.9, 17.4) |
| Ghana | 1998 | 41.8(34.1, 49.5) | 30.4(23.2, 37.6) | 27.8(20.8, 34.9) |
| Ghana | 2000 | 50.7(39.1, 62.2) | 24.7(14.7, 34.6) | 24.7(14.7, 34.6) |
| Ghana | 2003 | 54.8(47.0, 62.7) | 21.3(14.8, 27.8) | 23.9(17.1, 30.6) |
| Ghana | 2008 | 52.8(42.4, 63.2) | 29.2(19.7, 38.7) | 18.0(10.0, 26.0) |
| Ghana | 2014 | 61.9(52.6, 71.2) | 21.9(14.0, 29.9) | 16.2(9.1, 23.3) |
| Guatemala | 1987 | 38.0(33.3, 42.8) | 41.3(36.5, 46.2) | 20.7(16.7, 24.6) |
| Guatemala | 1995 | 43.7(36.3, 51.1) | 47.1(39.7, 54.6) | 9.2(4.9, 13.5) |
| Guatemala | 2014 | 56.2(49.2, 63.3) | 32.3(25.7, 38.9) | 11.5(6.9, 16.0) |
| Guinea | 1999 | 32.7(27.0, 38.4) | 33.1(27.4, 38.8) | 34.2(28.5, 40.0) |
| Guinea | 2005 | 34.6(29.4, 39.8) | 39.6(34.2, 45.0) | 25.8(21.0, 30.6) |
| Guinea | 2012 | 37.7(32.1, 43.2) | 33.6(28.1, 39.0) | 28.8(23.6, 34.0) |
| Guinea | 2018 | 35.3(29.8, 40.8) | 38.8(33.1, 44.4) | 26.0(20.9, 31.0) |
| Guyana | 2009 | 61.1(45.0, 77.3) | 36.1(20.2, 52.0) | 2.8(-2.7, 8.2) |
| Haiti | 1994 | 35.1(28.6, 41.6) | 37.0(30.4, 43.6) | 27.9(21.8, 34.0) |
| Haiti | 2000 | 30.6(25.5, 35.8) | 49.4(43.8, 54.9) | 20.0(15.5, 24.5) |
| Haiti | 2005 | 35.9(29.4, 42.5) | 43.2(36.4, 50.0) | 20.9(15.3, 26.4) |
| Haiti | 2012 | 42.1(35.7, 48.4) | 34.8(28.6, 40.9) | 23.2(17.7, 28.6) |
| Haiti | 2016 | 47.1(40.5, 53.7) | 34.5(28.3, 40.8) | 18.4(13.3, 23.5) |
| Honduras | 2005 | 64.2(54.5, 73.9) | 27.4(18.4, 36.4) | 8.4(2.8, 14.0) |
| Honduras | 2011 | 69.9(61.0, 78.8) | 19.4(11.7, 27.1) | 10.7(4.7, 16.7) |
| India | 1992 | 58.3(56.3, 60.3) | 28.4(26.6, 30.3) | 13.3(11.9, 14.7) |
| India | 1998 | 63.1(60.2, 66.0) | 28.0(25.3, 30.7) | 8.9(7.2, 10.7) |
| India | 2005 | 60.9(59.0, 62.8) | 25.8(24.1, 27.5) | 13.3(12.0, 14.6) |
| India | 2015 | 66.4(65.1, 67.7) | 23.3(22.1, 24.4) | 10.3(9.5, 11.1) |
| Indonesia | 1987 | 33.8(29.1, 38.4) | 49.7(44.8, 54.7) | 16.5(12.8, 20.2) |
| Indonesia | 1991 | 43.7(39.4, 48.1) | 42.7(38.4, 47.1) | 13.5(10.5, 16.5) |
| Indonesia | 1994 | 49.7(45.6, 53.9) | 31.9(28.0, 35.8) | 18.4(15.2, 21.6) |
| Indonesia | 1997 | 45.4(40.4, 50.4) | 45.4(40.4, 50.4) | 9.2(6.3, 12.2) |
| Indonesia | 2002 | 53.2(47.7, 58.7) | 34.2(28.9, 39.4) | 12.7(9.0, 16.3) |
| Indonesia | 2007 | 56.3(50.4, 62.2) | 34.1(28.4, 39.7) | 9.6(6.1, 13.2) |
| Indonesia | 2012 | 57.4(51.4, 63.4) | 34.6(28.8, 40.4) | 8.0(4.7, 11.3) |
| Indonesia | 2017 | 56.2(49.8, 62.6) | 27.5(21.7, 33.2) | 16.3(11.6, 21.1) |
| Jordan | 1990 | 65.5(58.4, 72.6) | 28.1(21.3, 34.8) | 6.4(2.7, 10.1) |
| Jordan | 1997 | 64.1(55.9, 72.4) | 23.7(16.4, 31.0) | 12.2(6.6, 17.8) |
| Jordan | 2002 | 69.9(59.3, 80.5) | 20.5(11.2, 29.9) | 9.6(2.8, 16.4) |
| Jordan | 2012 | 72.4(63.6, 81.3) | 11.2(4.9, 17.5) | 16.3(9.0, 23.7) |
| Jordan | 2017 | 64.2(54.5, 73.9) | 29.5(20.3, 38.7) | 6.3(1.4, 11.2) |
| Kenya | 1989 | 38.2(31.8, 44.7) | 41.9(35.4, 48.5) | 19.8(14.5, 25.1) |
| Kenya | 1993 | 31.3(25.2, 37.5) | 44.2(37.6, 50.9) | 24.4(18.7, 30.2) |
| Kenya | 1998 | 29.0(21.0, 37.1) | 55.6(46.9, 64.4) | 15.3(9.0, 21.7) |
| Kenya | 2003 | 35.7(30.2, 41.1) | 41.7(36.1, 47.3) | 22.7(17.9, 27.4) |
| Kenya | 2008 | 49.8(42.9, 56.6) | 30.0(23.7, 36.4) | 20.2(14.7, 25.7) |
| Kenya | 2014 | 48.7(44.2, 53.2) | 35.5(31.2, 39.9) | 15.7(12.4, 19.0) |
| Kyrgyzstan | 1997 | 63.8(49.9, 77.7) | 34.0(20.3, 47.7) | 2.1(-2.0, 6.3) |
| Kyrgyzstan | 2012 | 77.6(65.7, 89.4) | 14.3(4.4, 24.2) | 8.2(0.4, 15.9) |
| Lesotho | 2004 | 49.7(41.7, 57.6) | 42.5(34.6, 50.3) | 7.8(3.6, 12.1) |
| Lesotho | 2009 | 49.7(42.0, 57.3) | 36.4(29.0, 43.7) | 13.9(8.6, 19.2) |
| Lesotho | 2014 | 49.0(39.4, 58.7) | 31.7(22.7, 40.7) | 19.2(11.6, 26.8) |
| Liberia | 1986 | 43.2(36.6, 49.7) | 40.9(34.4, 47.4) | 15.9(11.1, 20.8) |
| Liberia | 2007 | 38.9(32.8, 45.1) | 41.0(34.8, 47.2) | 20.1(15.0, 25.1) |
| Liberia | 2013 | 38.3(31.7, 44.9) | 32.5(26.2, 38.9) | 29.2(23.0, 35.4) |
| Madagascar | 1992 | 31.9(27.8, 36.1) | 41.1(36.7, 45.5) | 26.9(23.0, 30.9) |
| Madagascar | 1997 | 40.8(34.3, 47.4) | 41.7(35.2, 48.3) | 17.4(12.4, 22.5) |
| Madagascar | 2003 | 45.2(39.5, 50.8) | 30.8(25.5, 36.0) | 24.1(19.2, 28.9) |
| Madagascar | 2008 | 42.6(37.4, 47.8) | 36.9(31.9, 42.0) | 20.5(16.2, 24.7) |
| Malawi | 1992 | 22.0(18.0, 26.0) | 51.3(46.5, 56.2) | 26.6(22.4, 30.9) |
| Malawi | 2000 | 30.0(27.1, 32.9) | 39.9(36.8, 42.9) | 30.2(27.3, 33.1) |
| Malawi | 2004 | 27.0(23.4, 30.6) | 45.7(41.6, 49.7) | 27.3(23.7, 30.9) |
| Malawi | 2010 | 35.8(32.7, 38.9) | 37.5(34.4, 40.6) | 26.7(23.9, 29.5) |
| Malawi | 2015 | 54.6(50.0, 59.2) | 26.5(22.4, 30.6) | 18.9(15.2, 22.5) |
| Mali | 1987 | 32.8(26.7, 38.8) | 29.3(23.4, 35.2) | 38.0(31.7, 44.3) |
| Mali | 1995 | 39.1(34.5, 43.6) | 38.4(33.9, 42.9) | 22.5(18.7, 26.4) |
| Mali | 2001 | 36.7(33.9, 39.5) | 29.3(26.6, 31.9) | 34.0(31.3, 36.8) |
| Mali | 2006 | 33.9(30.9, 36.8) | 32.4(29.5, 35.3) | 33.8(30.8, 36.7) |
| Mali | 2012 | 46.8(41.7, 52.0) | 27.1(22.6, 31.7) | 26.0(21.5, 30.5) |
| Mali | 2018 | 44.8(40.1, 49.6) | 24.4(20.3, 28.5) | 30.8(26.4, 35.1) |
| Morocco | 1987 | 49.6(45.2, 54.0) | 34.4(30.2, 38.6) | 16.0(12.8, 19.2) |
| Morocco | 1992 | 47.4(42.1, 52.7) | 37.2(32.1, 42.3) | 15.4(11.6, 19.2) |
| Morocco | 2003 | 72.4(63.6, 81.3) | 21.4(13.3, 29.6) | 6.1(1.4, 10.9) |
| Mozambique | 1997 | 32.6(26.9, 38.3) | 59.4(53.4, 65.4) | 8.0(4.7, 11.4) |
| Mozambique | 2003 | 32.4(28.7, 36.0) | 49.1(45.2, 52.9) | 18.6(15.6, 21.6) |
| Mozambique | 2011 | 37.6(33.0, 42.3) | 41.0(36.3, 45.7) | 21.3(17.4, 25.3) |
| Myanmar | 2015 | 53.2(43.1, 63.3) | 36.2(26.4, 45.9) | 10.6(4.4, 16.9) |
| Namibia | 1992 | 52.1(44.5, 59.6) | 31.4(24.3, 38.4) | 16.6(10.9, 22.2) |
| Namibia | 2000 | 37.0(27.5, 46.5) | 36.0(26.5, 45.5) | 27.0(18.3, 35.7) |
| Namibia | 2006 | 49.1(40.0, 58.3) | 32.8(24.2, 41.3) | 18.1(11.1, 25.1) |
| Namibia | 2013 | 35.9(26.6, 45.2) | 42.7(33.1, 52.3) | 21.4(13.4, 29.3) |
| Nepal | 1996 | 72.1(63.7, 80.5) | 23.4(15.5, 31.3) | 4.5(0.6, 8.4) |
| Nepal | 2001 | 53.8(47.9, 59.7) | 29.2(23.9, 34.6) | 17.0(12.5, 21.4) |
| Nepal | 2006 | 61.5(53.1, 69.9) | 26.9(19.3, 34.6) | 11.5(6.0, 17.1) |
| Nepal | 2011 | 73.0(65.5, 80.5) | 19.7(13.0, 26.4) | 7.3(2.9, 11.7) |
| Nepal | 2016 | 56.5(46.3, 66.7) | 37.0(27.0, 46.9) | 6.5(1.4, 11.6) |
| Nicaragua | 1998 | 40.2(31.2, 49.1) | 46.2(37.1, 55.2) | 13.7(7.4, 19.9) |
| Nicaragua | 2001 | 44.8(35.2, 54.3) | 33.3(24.3, 42.4) | 21.9(14.0, 29.9) |
| Niger | 1992 | 19.2(16.7, 21.7) | 33.9(30.9, 36.9) | 46.8(43.7, 50.0) |
| Niger | 1998 | 33.3(28.7, 37.8) | 39.8(35.1, 44.5) | 26.9(22.7, 31.2) |
| Niger | 2006 | 27.5(24.2, 30.7) | 32.2(28.9, 35.6) | 40.3(36.7, 43.9) |
| Niger | 2012 | 28.6(24.9, 32.2) | 28.1(24.4, 31.7) | 43.4(39.3, 47.4) |
| Nigeria | 1990 | 31.6(27.6, 35.5) | 29.1(25.2, 33.0) | 39.4(35.2, 43.5) |
| Nigeria | 2003 | 31.0(26.5, 35.4) | 30.5(26.1, 34.9) | 38.6(33.9, 43.2) |
| Nigeria | 2008 | 38.0(36.9, 39.2) | 26.3(25.2, 27.4) | 35.7(34.4, 37.0) |
| Nigeria | 2013 | 40.7(38.1, 43.3) | 29.9(27.4, 32.3) | 29.5(27.1, 31.9) |
| Nigeria | 2018 | 42.0(39.6, 44.4) | 24.0(21.9, 26.1) | 34.0(31.7, 36.4) |
| Pakistan | 1990 | 49.1(44.3, 54.0) | 34.7(30.1, 39.3) | 16.1(12.6, 19.7) |
| Pakistan | 2006 | 68.0(63.4, 72.6) | 22.8(18.7, 27.0) | 9.1(6.3, 12.0) |
| Pakistan | 2012 | 72.3(68.7, 75.8) | 19.3(16.2, 22.5) | 8.4(6.2, 10.6) |
| Pakistan | 2017 | 63.7(59.1, 68.3) | 28.0(23.7, 32.3) | 8.3(5.7, 11.0) |
| Papua New Guinea | 2017 | 49.1(42.5, 55.7) | 32.9(26.7, 39.1) | 18.0(13.0, 23.1) |
| Peru | 1986 | 37.6(32.1, 43.2) | 39.7(34.1, 45.3) | 22.7(17.9, 27.5) |
| Peru | 1991 | 37.3(30.7, 43.8) | 40.6(33.9, 47.2) | 22.2(16.6, 27.8) |
| Peru | 1996 | 52.8(47.0, 58.7) | 34.0(28.5, 39.6) | 13.1(9.2, 17.1) |
| Peru | 2000 | 50.0(43.3, 56.7) | 37.7(31.2, 44.3) | 12.3(7.8, 16.7) |
| Peru | 2004 | 54.1(45.6, 62.5) | 30.4(22.6, 38.2) | 15.6(9.4, 21.7) |
| Peru | 2007 | 54.1(45.6, 62.5) | 30.4(22.6, 38.2) | 15.6(9.4, 21.7) |
| Peru | 2009 | 46.0(36.8, 55.2) | 38.9(29.9, 48.0) | 15.0(8.4, 21.7) |
| Peru | 2010 | 48.6(36.8, 60.4) | 40.0(28.4, 51.6) | 11.4(3.9, 18.9) |
| Peru | 2011 | 40.7(28.0, 53.3) | 49.2(36.3, 62.0) | 10.2(2.4, 17.9) |
| Peru | 2012 | 59.4(47.7, 71.1) | 30.4(19.5, 41.4) | 10.1(3.0, 17.3) |
| Philippines | 1993 | 57.0(47.2, 66.8) | 31.0(21.9, 40.1) | 12.0(5.6, 18.4) |
| Philippines | 1998 | 47.4(36.1, 58.7) | 35.5(24.7, 46.4) | 17.1(8.6, 25.6) |
| Philippines | 2003 | 55.2(45.2, 65.2) | 28.1(19.1, 37.2) | 16.7(9.2, 24.2) |
| Philippines | 2008 | 66.2(54.8, 77.5) | 20.6(10.9, 30.3) | 13.2(5.1, 21.3) |
| Philippines | 2013 | 51.6(39.2, 63.9) | 32.8(21.2, 44.4) | 15.6(6.7, 24.6) |
| Philippines | 2017 | 61.0(52.2, 69.9) | 27.1(19.1, 35.2) | 11.9(6.0, 17.7) |
| Rwanda | 1992 | 34.7(30.6, 38.9) | 38.4(34.2, 42.6) | 26.8(23.0, 30.7) |
| Rwanda | 2000 | 28.0(24.6, 31.3) | 41.9(38.3, 45.6) | 30.1(26.7, 33.5) |
| Rwanda | 2005 | 33.1(28.9, 37.2) | 40.7(36.4, 45.0) | 26.3(22.4, 30.1) |
| Rwanda | 2010 | 44.6(38.9, 50.2) | 37.6(32.2, 43.1) | 17.8(13.5, 22.1) |
| Rwanda | 2014 | 47.5(40.1, 54.8) | 31.3(24.5, 38.1) | 21.2(15.2, 27.2) |
| Sao Tome and Principe | 2008 | 46.5(31.4, 61.6) | 30.2(16.3, 44.1) | 23.3(10.5, 36.0) |
| Senegal | 1986 | 33.9(30.0, 37.7) | 27.8(24.2, 31.5) | 38.3(34.4, 42.3) |
| Senegal | 1992 | 37.0(32.9, 41.2) | 32.1(28.0, 36.1) | 30.9(26.9, 34.9) |
| Senegal | 1997 | 37.5(32.1, 43.0) | 27.2(22.2, 32.3) | 35.2(29.8, 40.6) |
| Senegal | 2005 | 44.9(39.8, 50.0) | 27.5(22.9, 32.2) | 27.5(22.9, 32.2) |
| Senegal | 2010 | 56.8(50.1, 63.5) | 26.8(20.8, 32.7) | 16.4(11.4, 21.4) |
| Senegal | 2012 | 52.8(44.6, 61.1) | 31.7(24.0, 39.4) | 15.5(9.5, 21.5) |
| Senegal | 2014 | 52.8(46.1, 59.4) | 34.9(28.5, 41.2) | 12.4(8.0, 16.8) |
| Senegal | 2015 | 54.4(43.4, 65.5) | 38.0(27.2, 48.7) | 7.6(1.7, 13.5) |
| Senegal | 2016 | 52.5(41.5, 63.5) | 40.0(29.2, 50.8) | 7.5(1.7, 13.3) |
| Senegal | 2017 | 62.9(56.3, 69.6) | 25.9(19.8, 31.9) | 11.2(6.9, 15.6) |
| Sierra Leone | 2008 | 34.6(29.2, 40.0) | 44.3(38.6, 49.9) | 21.1(16.5, 25.8) |
| Sierra Leone | 2013 | 35.2(31.8, 38.5) | 41.5(38.0, 45.0) | 23.3(20.3, 26.3) |
| Tajikistan | 2012 | 49.1(39.7, 58.5) | 36.4(27.3, 45.4) | 14.5(7.9, 21.2) |
| Tajikistan | 2017 | 44.1(34.9, 53.4) | 44.1(34.9, 53.4) | 11.7(5.7, 17.7) |
| Tanzania | 1991 | 36.4(31.8, 41.0) | 44.8(40.0, 49.5) | 18.8(15.1, 22.6) |
| Tanzania | 1996 | 31.2(26.3, 36.2) | 45.2(39.9, 50.6) | 23.5(19.0, 28.1) |
| Tanzania | 1999 | 34.2(28.1, 40.2) | 43.9(37.6, 50.2) | 21.9(16.7, 27.2) |
| Tanzania | 2004 | 40.7(35.9, 45.5) | 37.7(33.0, 42.5) | 21.6(17.6, 25.6) |
| Tanzania | 2010 | 39.1(33.3, 44.8) | 38.3(32.6, 44.1) | 22.6(17.7, 27.6) |
| Tanzania | 2015 | 46.6(40.6, 52.6) | 31.2(25.6, 36.8) | 22.2(17.2, 27.2) |
| Timor-Leste | 2009 | 41.1(34.6, 47.5) | 37.9(31.6, 44.3) | 21.0(15.6, 26.3) |
| Timor-Leste | 2016 | 52.7(44.1, 61.4) | 23.3(15.9, 30.6) | 24.0(16.6, 31.4) |
| Togo | 1988 | 36.6(31.4, 41.8) | 31.4(26.4, 36.4) | 32.0(27.0, 37.1) |
| Togo | 1998 | 61.0(52.2, 69.9) | 30.5(22.2, 38.9) | 8.5(3.4, 13.5) |
| Togo | 2013 | 47.7(40.3, 55.1) | 27.6(20.9, 34.2) | 24.7(18.3, 31.1) |
| Turkey | 1993 | 51.9(43.3, 60.5) | 38.2(29.8, 46.5) | 9.9(4.8, 15.1) |
| Turkey | 1998 | 57.8(47.1, 68.5) | 30.1(20.2, 40.1) | 12.0(5.0, 19.1) |
| Turkey | 2003 | 53.7(40.3, 67.1) | 27.8(15.7, 39.8) | 18.5(8.1, 29.0) |
| Turkey | 2008 | 68.0(49.3, 86.7) | 16.0(1.3, 30.7) | 16.0(1.3, 30.7) |
| Uganda | 1988 | 32.0(26.3, 37.8) | 36.0(30.0, 41.9) | 32.0(26.3, 37.8) |
| Uganda | 1995 | 24.6(20.1, 29.0) | 48.9(43.7, 54.1) | 26.5(22.0, 31.1) |
| Uganda | 2000 | 28.4(24.6, 32.1) | 45.4(41.3, 49.6) | 26.2(22.6, 29.9) |
| Uganda | 2006 | 30.7(25.8, 35.6) | 39.5(34.3, 44.7) | 29.8(25.0, 34.7) |
| Uganda | 2011 | 39.9(34.5, 45.4) | 33.2(28.0, 38.5) | 26.8(21.9, 31.8) |
| Uganda | 2016 | 52.7(47.3, 58.2) | 28.7(23.8, 33.6) | 18.6(14.4, 22.8) |
| Yemen | 1991 | 38.5(34.3, 42.7) | 40.0(35.8, 44.3) | 21.5(17.9, 25.0) |
| Yemen | 2013 | 59.3(54.2, 64.3) | 29.2(24.6, 33.9) | 11.5(8.2, 14.7) |
| Zambia | 1992 | 30.0(26.5, 33.4) | 39.3(35.6, 43.0) | 30.7(27.2, 34.2) |
| Zambia | 1996 | 23.8(19.7, 28.0) | 45.0(40.1, 49.8) | 31.2(26.7, 35.7) |
| Zambia | 2001 | 31.3(26.9, 35.7) | 34.4(29.8, 38.9) | 34.4(29.8, 38.9) |
| Zambia | 2007 | 40.9(35.2, 46.7) | 31.3(25.9, 36.7) | 27.8(22.5, 33.0) |
| Zambia | 2013 | 43.4(38.5, 48.2) | 33.1(28.5, 37.7) | 23.6(19.4, 27.7) |
| Zimbabwe | 1988 | 45.9(38.8, 52.9) | 34.0(27.3, 40.7) | 20.1(14.4, 25.8) |
| Zimbabwe | 1994 | 29.5(20.0, 39.1) | 47.7(37.2, 58.2) | 22.7(13.9, 31.5) |
| Zimbabwe | 1999 | 36.3(27.8, 44.8) | 36.3(27.8, 44.8) | 27.4(19.5, 35.3) |
| Zimbabwe | 2005 | 36.2(28.2, 44.3) | 46.4(38.0, 54.7) | 17.4(11.0, 23.7) |
| Zimbabwe | 2010 | 46.0(39.2, 52.7) | 34.1(27.7, 40.5) | 19.9(14.5, 25.3) |
| Zimbabwe | 2015 | 50.5(43.8, 57.1) | 30.1(24.0, 36.2) | 19.4(14.2, 24.7) |

**Note:**

1. Countries with less than a total of 20 under-five deaths were excluded from the analysis

**Additional Table 2. Share of neonatal, postneonatal, and childhood to total under-5 deaths at aggregate-level, latest survey rounds between 2008 and 2018**

|  | **Neonatal** | **Post-neonatal** | **Childhood** |
| --- | --- | --- | --- |
| **All available countries** | 52.5(52.2, 52.9) | 28.6(28.3, 29.0) | 18.9(18.6, 19.3) |
| ***By income class*** |  |  |  |
| Low-income countries | 44.2(43.7, 44.7) | 32.8(32.3, 33.3) | 23.0(22.5, 23.5) |
| Lower-middle-income countries | 56.2(55.8, 56.6) | 26.6(26.2, 27.0) | 17.2(16.8, 17.6) |
| Upper-middle-income countries | 54.7(53.8, 55.5) | 33.0(32.2, 33.9) | 12.3(11.6, 13.0) |

**Additional Table 3. Share of neonatal, postneonatal, and childhood to total under-5 deaths at aggregate-level including the countries with 0 recorded deaths in any period of life, latest survey rounds**

|  | **Neonatal** | **Post-neonatal** | **Childhood** |
| --- | --- | --- | --- |
| **All available countries** | 52.6(52.1, 53.1) | 28.1(27.7, 28.6) | 19.3(19.0, 19.7) |
| ***By income class*** |  |  |  |
| Low-income countries | 43.8(43.3, 44.3) | 32.8(32.3, 33.3) | 23.4(22.8, 24.0) |
| Lower-middle-income countries | 56.6(56.1, 57.1) | 26.7(26.2, 27.3) | 16.7(16.0, 17.4) |
| Upper-middle-income countries | 54.7(53.8, 55.5) | 33.0(32.2, 33.9) | 12.3(11.6, 13.0) |

**Additional Table 4. Change in share of neonatal to total under-5 deaths**

| **Country** | **Year range** | **Share of neonatal to total under-5 deaths (%)** | | **Total change in the share (percentage points)** |
| --- | --- | --- | --- | --- |
|  |  | **Earliest year** | **Latest year** |  |
| Bangladesh | 1993-2014 | 57.9(51.0, 64.9) | 67.5(60.1, 75.0) | 9.6(-0.7, 19.8) |
| Benin | 1996-2017 | 44.4(34.1, 54.8) | 43.0(38.5, 47.5) | -1.4(-12.6, 9.8) |
| Bolivia | 1989-2008 | 35.7(30.6, 40.8) | 55.4(49.2, 61.5) | 19.7(11.7, 27.7) |
| Burkina Faso | 1993-2010 | 33.0(29.0, 37.0) | 31.0(27.5, 34.4) | -2.0(-7.3, 3.3) |
| Burundi | 1987-2016 | 35.5(30.3, 40.8) | 38.1(33.7, 42.6) | 2.6(-4.3, 9.5) |
| Cambodia | 2000-2014 | 35.8(31.3, 40.4) | 64.0(55.2, 72.9) | 28.2(18.3, 38.2) |
| Cameroon | 1991-2011 | 33.7(26.5, 41.0) | 35.8(31.6, 40.0) | 2.0(-6.4, 10.5) |
| Chad | 1996-2014 | 33.7(30.0, 37.4) | 35.6(32.6, 38.6) | 1.9(-2.9, 6.7) |
| Comoros | 1996-2012 | 55.1(44.7, 65.4) | 54.5(43.4, 65.7) | -0.5(-15.9, 14.9) |
| Congo | 2005-2011 | 34.5(28.4, 40.5) | 44.1(37.5, 50.7) | 9.6(0.7, 18.6) |
| Democratic Republic of the Congo | 2007-2013 | 40.1(36.0, 44.3) | 37.6(34.0, 41.2) | -2.5(-8.0, 2.9) |
| Dominican Republic | 1986-2013 | 62.9(55.7, 70.2) | 82.8(73.0, 92.6) | 19.8(6.0, 33.6) |
| Egypt | 1988-2014 | 47.3(43.2, 51.4) | 55.2(48.6, 61.7) | 7.9(0.1, 15.6) |
| Ethiopia | 2000-2016 | 39.2(36.3, 42.2) | 51.1(46.3, 55.8) | 11.8(6.3, 17.4) |
| Gabon | 2000-2012 | 50.7(39.1, 62.2) | 51.3(43.3, 59.3) | 0.6(-13.5, 14.7) |
| Ghana | 1988-2014 | 40.4(35.8, 44.9) | 61.9(52.6, 71.2) | 21.5(11.1, 32.0) |
| Guatemala | 1987-2014 | 38.0(33.3, 42.8) | 56.2(49.2, 63.3) | 18.2(9.8, 26.7) |
| Guinea | 1999-2018 | 32.7(27.0, 38.4) | 35.3(29.8, 40.8) | 2.6(-5.3, 10.5) |
| Haiti | 1994-2016 | 35.1(28.6, 41.6) | 47.1(40.5, 53.7) | 12.0(2.7, 21.3) |
| Honduras | 2005-2011 | 64.2(54.5, 73.9) | 69.9(61.0, 78.8) | 5.7(-7.5, 18.9) |
| India | 1992-2015 | 58.3(56.3, 60.3) | 66.4(65.1, 67.7) | 8.1(5.8, 10.5) |
| Indonesia | 1987-2017 | 33.8(29.1, 38.4) | 56.2(49.8, 62.6) | 22.5(14.6, 30.3) |
| Jordan | 1990-2017 | 65.5(58.4, 72.6) | 64.2(54.5, 73.9) | -1.3(-13.3, 10.8) |
| Kenya | 1989-2014 | 38.2(31.8, 44.7) | 48.7(44.2, 53.2) | 10.5(2.5, 18.5) |
| Kyrgyzstan | 1997-2012 | 63.8(49.9, 77.7) | 77.6(65.7, 89.4) | 13.7(-4.7, 32.1) |
| Lesotho | 2004-2014 | 49.7(41.7, 57.6) | 49.0(39.4, 58.7) | -0.6(-13.2, 11.9) |
| Liberia | 1986-2013 | 43.2(36.6, 49.7) | 38.3(31.7, 44.9) | -4.9(-14.2, 4.4) |
| Madagascar | 1992-2008 | 31.9(27.8, 36.1) | 42.6(37.4, 47.8) | 10.7(4.1, 17.3) |
| Malawi | 1992-2015 | 22.0(18.0, 26.0) | 54.6(50.0, 59.2) | 32.6(26.4, 38.7) |
| Mali | 1987-2018 | 32.8(26.7, 38.8) | 44.8(40.1, 49.6) | 12.1(4.2, 19.9) |
| Morocco | 1987-2003 | 49.6(45.2, 54.0) | 72.4(63.6, 81.3) | 22.8(12.2, 33.5) |
| Mozambique | 1997-2011 | 32.6(26.9, 38.3) | 37.6(33.0, 42.3) | 5.1(-2.3, 12.5) |
| Namibia | 1992-2013 | 52.1(44.5, 59.6) | 35.9(26.6, 45.2) | -16.1(-28.3, -4.0) |
| Nepal | 1996-2016 | 72.1(63.7, 80.5) | 56.5(46.3, 66.7) | -15.6(-28.7, -2.4) |
| Niger | 1992-2012 | 19.2(16.7, 21.7) | 28.6(24.9, 32.2) | 9.3(5.0, 13.7) |
| Nigeria | 1990-2018 | 31.6(27.6, 35.5) | 42.0(39.6, 44.4) | 10.5(5.7, 15.3) |
| Pakistan | 1990-2017 | 49.1(44.3, 54.0) | 63.7(59.1, 68.3) | 14.5(7.8, 21.2) |
| Peru | 1986-2012 | 37.6(32.1, 43.2) | 59.4(47.7, 71.1) | 21.8(9.0, 34.6) |
| Philippines | 1993-2017 | 57.0(47.2, 66.8) | 61.0(52.2, 69.9) | 4.0(-9.2, 17.2) |
| Rwanda | 1992-2014 | 34.7(30.6, 38.9) | 47.5(40.1, 54.8) | 12.7(4.5, 21.0) |
| Senegal | 1986-2017 | 33.9(30.0, 37.7) | 62.9(56.3, 69.6) | 29.1(21.5, 36.7) |
| Sierra Leone | 2008-2013 | 34.6(29.2, 40.0) | 35.2(31.8, 38.5) | 0.6(-5.8, 7.0) |
| Tajikistan | 2012-2017 | 49.1(39.7, 58.5) | 44.1(34.9, 53.4) | -4.9(-18.2, 8.3) |
| Tanzania | 1991-2015 | 36.4(31.8, 41.0) | 46.6(40.6, 52.6) | 10.2(2.7, 17.7) |
| Timor-Leste | 2009-2016 | 41.1(34.6, 47.5) | 52.7(44.1, 61.4) | 11.6(0.9, 22.4) |
| Togo | 1988-2013 | 36.6(31.4, 41.8) | 47.7(40.3, 55.1) | 11.1(2.2, 20.1) |
| Uganda | 1988-2016 | 32.0(26.3, 37.8) | 52.7(47.3, 58.2) | 20.7(12.7, 28.7) |
| Yemen | 1991-2013 | 38.5(34.3, 42.7) | 59.3(54.2, 64.3) | 20.8(14.2, 27.4) |
| Zambia | 1992-2013 | 30.0(26.5, 33.4) | 43.4(38.5, 48.2) | 13.4(7.5, 19.2) |
| Zimbabwe | 1988-2015 | 45.9(38.8, 52.9) | 50.5(43.8, 57.1) | 4.6(-5.1, 14.3) |

**Note:**

1. Countries with less than a total of 20 under-five deaths were excluded from the analysis
2. Countries with the latest year conducted earlier than 2000 were excluded from the analysis

**Additional Table 5. Change in share of postneonatal to total under-5 deaths**

| **Country** | **Year range** | **Share of postneonatal to total under-5 deaths (%)** | | **Total change in the share (percentage points)** |
| --- | --- | --- | --- | --- |
|  |  | **Earliest year** | **Latest year** |  |
| Bangladesh | 1993-2014 | 30.8(24.3, 37.3) | 23.4(16.7, 30.1) | -7.4(-16.9, 2.1) |
| Benin | 1996-2017 | 34.4(24.6, 44.3) | 29.9(25.7, 34.0) | -4.6(-15.0, 5.9) |
| Bolivia | 1989-2008 | 38.3(33.1, 43.5) | 36.7(30.7, 42.6) | -1.7(-9.6, 6.3) |
| Burkina Faso | 1993-2010 | 33.8(29.7, 37.8) | 36.2(32.6, 39.7) | 2.4(-3.0, 7.8) |
| Burundi | 1987-2016 | 31.2(26.1, 36.2) | 37.0(32.6, 41.5) | 5.9(-0.9, 12.7) |
| Cambodia | 2000-2014 | 49.4(44.7, 54.2) | 25.4(17.4, 33.5) | -24.0(-34.1, -13.9) |
| Cameroon | 1991-2011 | 28.2(21.3, 35.2) | 31.0(27.0, 35.1) | 2.8(-5.4, 10.9) |
| Chad | 1996-2014 | 35.8(32.0, 39.5) | 33.7(30.7, 36.7) | -2.0(-6.8, 2.7) |
| Comoros | 1996-2012 | 33.7(23.8, 43.6) | 28.6(18.4, 38.7) | -5.1(-19.5, 9.2) |
| Congo | 2005-2011 | 46.6(40.3, 53.0) | 26.8(21.0, 32.7) | -19.8(-28.5, -11.1) |
| Democratic Republic of the Congo | 2007-2013 | 37.4(33.4, 41.5) | 36.3(32.7, 39.9) | -1.1(-6.5, 4.3) |
| Dominican Republic | 1986-2013 | 23.5(17.1, 29.9) | 5.2(-0.6, 10.9) | -18.4(-29.9, -6.8) |
| Egypt | 1988-2014 | 36.8(32.9, 40.8) | 35.0(28.7, 41.3) | -1.8(-9.3, 5.6) |
| Ethiopia | 2000-2016 | 35.5(32.7, 38.4) | 30.4(26.0, 34.8) | -5.1(-10.5, 0.2) |
| Gabon | 2000-2012 | 24.7(14.7, 34.6) | 21.7(15.1, 28.3) | -2.9(-14.7, 8.8) |
| Ghana | 1988-2014 | 26.7(22.6, 30.8) | 21.9(14.0, 29.9) | -4.8(-14.1, 4.6) |
| Guatemala | 1987-2014 | 41.3(36.5, 46.2) | 32.3(25.7, 38.9) | -9.0(-17.4, -0.6) |
| Guinea | 1999-2018 | 33.1(27.4, 38.8) | 38.8(33.1, 44.4) | 5.7(-2.4, 13.7) |
| Haiti | 1994-2016 | 37.0(30.4, 43.6) | 34.5(28.3, 40.8) | -2.5(-11.6, 6.6) |
| Honduras | 2005-2011 | 27.4(18.4, 36.4) | 19.4(11.7, 27.1) | -8.0(-19.8, 3.9) |
| India | 1992-2015 | 28.4(26.6, 30.3) | 23.3(22.1, 24.4) | -5.2(-7.3, -3.1) |
| Indonesia | 1987-2017 | 49.7(44.8, 54.7) | 27.5(21.7, 33.2) | -22.3(-30.1, -14.5) |
| Jordan | 1990-2017 | 28.1(21.3, 34.8) | 29.5(20.3, 38.7) | 1.4(-10.0, 12.8) |
| Kenya | 1989-2014 | 41.9(35.4, 48.5) | 35.5(31.2, 39.9) | -6.4(-14.2, 1.4) |
| Kyrgyzstan | 1997-2012 | 34.0(20.3, 47.7) | 14.3(4.4, 24.2) | -19.8(-36.8, -2.7) |
| Lesotho | 2004-2014 | 42.5(34.6, 50.3) | 31.7(22.7, 40.7) | -10.8(-22.9, 1.4) |
| Liberia | 1986-2013 | 40.9(34.4, 47.4) | 32.5(26.2, 38.9) | -8.4(-17.5, 0.8) |
| Madagascar | 1992-2008 | 41.1(36.7, 45.5) | 36.9(31.9, 42.0) | -4.2(-10.9, 2.5) |
| Malawi | 1992-2015 | 51.3(46.5, 56.2) | 26.5(22.4, 30.6) | -24.8(-31.1, -18.5) |
| Mali | 1987-2018 | 29.3(23.4, 35.2) | 24.4(20.3, 28.5) | -4.8(-11.9, 2.2) |
| Morocco | 1987-2003 | 34.4(30.2, 38.6) | 21.4(13.3, 29.6) | -13.0(-23.1, -2.9) |
| Mozambique | 1997-2011 | 59.4(53.4, 65.4) | 41.0(36.3, 45.7) | -18.4(-26.0, -10.8) |
| Namibia | 1992-2013 | 31.4(24.3, 38.4) | 42.7(33.1, 52.3) | 11.4(-0.4, 23.1) |
| Nepal | 1996-2016 | 23.4(15.5, 31.3) | 37.0(27.0, 46.9) | 13.5(0.9, 26.1) |
| Niger | 1992-2012 | 33.9(30.9, 36.9) | 28.1(24.4, 31.7) | -5.9(-10.7, -1.1) |
| Nigeria | 1990-2018 | 29.1(25.2, 33.0) | 24.0(21.9, 26.1) | -5.1(-9.4, -0.8) |
| Pakistan | 1990-2017 | 34.7(30.1, 39.3) | 28.0(23.7, 32.3) | -6.7(-13.0, -0.4) |
| Peru | 1986-2012 | 39.7(34.1, 45.3) | 30.4(19.5, 41.4) | -9.2(-22.0, 3.5) |
| Philippines | 1993-2017 | 31.0(21.9, 40.1) | 27.1(19.1, 35.2) | -3.9(-16.1, 8.3) |
| Rwanda | 1992-2014 | 38.4(34.2, 42.6) | 31.3(24.5, 38.1) | -7.1(-15.3, 1.1) |
| Senegal | 1986-2017 | 27.8(24.2, 31.5) | 25.9(19.8, 31.9) | -2.0(-9.1, 5.2) |
| Sierra Leone | 2008-2013 | 44.3(38.6, 49.9) | 41.5(38.0, 45.0) | -2.8(-9.4, 3.9) |
| Tajikistan | 2012-2017 | 36.4(27.3, 45.4) | 44.1(34.9, 53.4) | 7.8(-5.2, 20.8) |
| Tanzania | 1991-2015 | 44.8(40.0, 49.5) | 31.2(25.6, 36.8) | -13.6(-21.0, -6.1) |
| Timor-Leste | 2009-2016 | 37.9(31.6, 44.3) | 23.3(15.9, 30.6) | -14.7(-24.8, -4.6) |
| Togo | 1988-2013 | 31.4(26.4, 36.4) | 27.6(20.9, 34.2) | -3.8(-12.3, 4.6) |
| Uganda | 1988-2016 | 36.0(30.0, 41.9) | 28.7(23.8, 33.6) | -7.3(-15.0, 0.3) |
| Yemen | 1991-2013 | 40.0(35.8, 44.3) | 29.2(24.6, 33.9) | -10.8(-17.2, -4.4) |
| Zambia | 1992-2013 | 39.3(35.6, 43.0) | 33.1(28.5, 37.7) | -6.2(-12.2, -0.2) |
| Zimbabwe | 1988-2015 | 34.0(27.3, 40.7) | 30.1(24.0, 36.2) | -3.9(-13.0, 5.2) |

**Note:**

1. Countries with less than a total of 20 under-five deaths were excluded from the analysis
2. Countries with the latest year conducted earlier than 2000 were excluded from the analysis

**Additional Table 6. Change in share of childhood to total under-5 deaths**

| **Country** | **Year range** | **Share of childhood to total under-5 deaths (%)** | | **Total change in the share (percentage points)** |
| --- | --- | --- | --- | --- |
|  |  | **Earliest year** | **Latest year** |  |
| Bangladesh | 1993-2014 | 11.3(6.8, 15.7) | 9.1(4.5, 13.6) | -2.2(-8.7, 4.3) |
| Benin | 1996-2017 | 21.1(12.6, 29.6) | 27.1(23.1, 31.1) | 6.0(-3.9, 15.9) |
| Bolivia | 1989-2008 | 26.0(21.4, 30.7) | 8.0(4.6, 11.3) | -18.1(-24.2, -11.9) |
| Burkina Faso | 1993-2010 | 33.2(29.2, 37.2) | 32.9(29.4, 36.3) | -0.4(-5.7, 5.0) |
| Burundi | 1987-2016 | 33.3(28.2, 38.5) | 24.8(20.9, 28.8) | -8.5(-14.9, -2.1) |
| Cambodia | 2000-2014 | 14.8(11.4, 18.1) | 10.5(4.9, 16.2) | -4.2(-11.4, 2.9) |
| Cameroon | 1991-2011 | 38.0(30.6, 45.5) | 33.2(29.1, 37.3) | -4.8(-13.2, 3.6) |
| Chad | 1996-2014 | 30.5(26.9, 34.1) | 30.7(27.8, 33.6) | 0.2(-4.5, 4.8) |
| Comoros | 1996-2012 | 11.2(4.6, 17.8) | 16.9(8.5, 25.3) | 5.6(-5.0, 16.3) |
| Congo | 2005-2011 | 18.9(13.9, 23.9) | 29.1(23.1, 35.1) | 10.2(2.4, 18.0) |
| Democratic Republic of the Congo | 2007-2013 | 22.4(18.9, 25.9) | 26.1(22.8, 29.4) | 3.6(-1.2, 8.5) |
| Dominican Republic | 1986-2013 | 13.5(8.4, 18.7) | 12.1(3.6, 20.5) | -1.5(-11.6, 8.7) |
| Egypt | 1988-2014 | 15.9(12.9, 18.9) | 9.9(5.9, 13.8) | -6.0(-11.4, -0.6) |
| Ethiopia | 2000-2016 | 25.2(22.6, 27.8) | 18.5(14.8, 22.2) | -6.7(-11.5, -1.9) |
| Gabon | 2000-2012 | 24.7(14.7, 34.6) | 27.0(19.9, 34.1) | 2.3(-10.1, 14.7) |
| Ghana | 1988-2014 | 33.0(28.6, 37.3) | 16.2(9.1, 23.3) | -16.8(-26.4, -7.1) |
| Guatemala | 1987-2014 | 20.7(16.7, 24.6) | 11.5(6.9, 16.0) | -9.2(-15.7, -2.6) |
| Guinea | 1999-2018 | 34.2(28.5, 40.0) | 26.0(20.9, 31.0) | -8.3(-15.9, -0.6) |
| Haiti | 1994-2016 | 27.9(21.8, 34.0) | 18.4(13.3, 23.5) | -9.5(-17.4, -1.6) |
| Honduras | 2005-2011 | 8.4(2.8, 14.0) | 10.7(4.7, 16.7) | 2.3(-6.0, 10.6) |
| India | 1992-2015 | 13.3(11.9, 14.7) | 10.3(9.5, 11.1) | -3.0(-4.5, -1.4) |
| Indonesia | 1987-2017 | 16.5(12.8, 20.2) | 16.3(11.6, 21.1) | -0.2(-6.2, 5.8) |
| Jordan | 1990-2017 | 6.4(2.7, 10.1) | 6.3(1.4, 11.2) | -0.1(-6.3, 6.1) |
| Kenya | 1989-2014 | 19.8(14.5, 25.1) | 15.7(12.4, 19.0) | -4.1(-10.1, 2.0) |
| Kyrgyzstan | 1997-2012 | 2.1(-2.0, 6.3) | 8.2(0.4, 15.9) | 6.0(-3.0, 15.1) |
| Lesotho | 2004-2014 | 7.8(3.6, 12.1) | 19.2(11.6, 26.8) | 11.4(3.2, 19.6) |
| Liberia | 1986-2013 | 15.9(11.1, 20.8) | 29.2(23.0, 35.4) | 13.3(5.4, 21.1) |
| Madagascar | 1992-2008 | 26.9(23.0, 30.9) | 20.5(16.2, 24.7) | -6.5(-12.4, -0.6) |
| Malawi | 1992-2015 | 26.6(22.4, 30.9) | 18.9(15.2, 22.5) | -7.8(-13.4, -2.2) |
| Mali | 1987-2018 | 38.0(31.7, 44.3) | 30.8(26.4, 35.1) | -7.2(-14.8, 0.3) |
| Morocco | 1987-2003 | 16.0(12.8, 19.2) | 6.1(1.4, 10.9) | -9.9(-17.5, -2.3) |
| Mozambique | 1997-2011 | 8.0(4.7, 11.4) | 21.3(17.4, 25.3) | 13.3(7.7, 18.9) |
| Namibia | 1992-2013 | 16.6(10.9, 22.2) | 21.4(13.4, 29.3) | 4.8(-4.8, 14.3) |
| Nepal | 1996-2016 | 4.5(0.6, 8.4) | 6.5(1.4, 11.6) | 2.0(-4.3, 8.3) |
| Niger | 1992-2012 | 46.8(43.7, 50.0) | 43.4(39.3, 47.4) | -3.5(-8.6, 1.7) |
| Nigeria | 1990-2018 | 39.4(35.2, 43.5) | 34.0(31.7, 36.4) | -5.3(-10.0, -0.6) |
| Pakistan | 1990-2017 | 16.1(12.6, 19.7) | 8.3(5.7, 11.0) | -7.8(-12.3, -3.4) |
| Peru | 1986-2012 | 22.7(17.9, 27.5) | 10.1(3.0, 17.3) | -12.6(-23.1, -2.0) |
| Philippines | 1993-2017 | 12.0(5.6, 18.4) | 11.9(6.0, 17.7) | -0.1(-8.9, 8.6) |
| Rwanda | 1992-2014 | 26.8(23.0, 30.7) | 21.2(15.2, 27.2) | -5.6(-13.0, 1.8) |
| Senegal | 1986-2017 | 38.3(34.4, 42.3) | 11.2(6.9, 15.6) | -27.1(-34.3, -20.0) |
| Sierra Leone | 2008-2013 | 21.1(16.5, 25.8) | 23.3(20.3, 26.3) | 2.2(-3.4, 7.8) |
| Tajikistan | 2012-2017 | 14.5(7.9, 21.2) | 11.7(5.7, 17.7) | -2.8(-11.8, 6.2) |
| Tanzania | 1991-2015 | 18.8(15.1, 22.6) | 22.2(17.2, 27.2) | 3.4(-2.8, 9.5) |
| Timor-Leste | 2009-2016 | 21.0(15.6, 26.3) | 24.0(16.6, 31.4) | 3.0(-6.0, 12.1) |
| Togo | 1988-2013 | 32.0(27.0, 37.1) | 24.7(18.3, 31.1) | -7.3(-15.7, 1.1) |
| Uganda | 1988-2016 | 32.0(26.3, 37.8) | 18.6(14.4, 22.8) | -13.4(-20.4, -6.4) |
| Yemen | 1991-2013 | 21.5(17.9, 25.0) | 11.5(8.2, 14.7) | -10.0(-15.1, -5.0) |
| Zambia | 1992-2013 | 30.7(27.2, 34.2) | 23.6(19.4, 27.7) | -7.2(-12.7, -1.6) |
| Zimbabwe | 1988-2015 | 20.1(14.4, 25.8) | 19.4(14.2, 24.7) | -0.7(-8.4, 7.1) |

**Note:**

1. Countries with less than a total of 20 under-five deaths were excluded from the analysis
2. Countries with the latest year conducted earlier than 2000 were excluded from the analysis
